# Arteriovenous Malformation of the Pancreas

**DOI:** 10.1155/2011/612657

**Published:** 2011-07-06

**Authors:** Alexandros Charalabopoulos, Nikolas Macheras, Sylvia Krivan, Konstantinos Petropoulos, Evangelos Misiakos, Anastasios Macheras

**Affiliations:** 3rd Department of Surgery, Attikon University Hospital, University of Athens School of Medicine, 1 Rimini Street, Chaidari, 124 62 Athens, Greece

## Abstract

Pancreatic arteriovenous malformation (PAVM) is a very rare and mostly congenital lesion, with less than 80 cases described in the English-published literature. It is defined as a tumorous vascular abnormality that is constructed between an anomalous bypass anastomosis of the arterial and venous networks within the pancreas. It represents about 5% of all arteriovenous malformations found in the gastrointestinal tract. Herein, we present a 64-year-old patient with symptomatic PAVM involving the body and tail of the organ, which was successfully treated by transcatheter arterial embolization. The disease spectrum and review of the literature are also presented.

## 1. Introduction

Pancreatic arteriovenous malformation (PAVM) is a rare condition with various clinical manifestations. In most cases, the condition is congenital and the diagnosis is accidental since most of the patients are asymptomatic and the tumorous mass is revealed in an imaging investigation performed for a different reason. The widespread use of imaging techniques such as color Doppler sonography, contrast-enhanced computed tomography, magnetic resonance imaging, and angiography is the exact reason why an increase in the number of pancreatic AVM cases has been reported in recent years. Nevertheless, some present with symptoms varying from abdominal pain, gastrointestinal bleeding, jaundice, and more. We report a case of a symptomatic pancreatic arteriovenous malformation in a 64-year-old woman, which was successfully treated by transarterial embolisation.

## 2. Case Presentation

**Figure 1:**
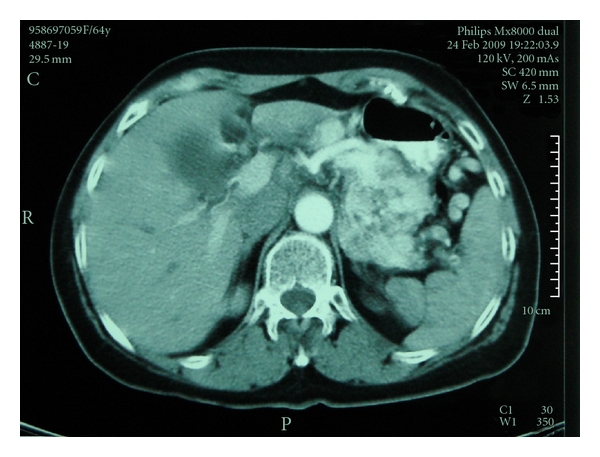
Contrast-enhanced axial CT scan through the pancreas shows a large hypervascular mass in the pancreatic body and tail. Note the presence of dilated perisplenic vessels.

**Figure 2:**
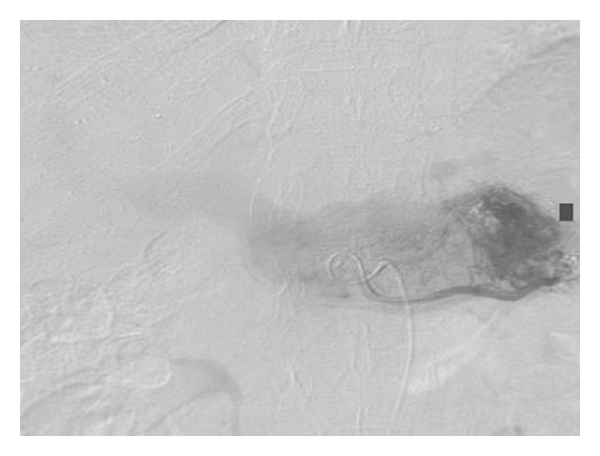
Superior mesenteric angiogram shows prominent vascularity in the pancreatic mass during the arterial phase.

**Figure 3:**
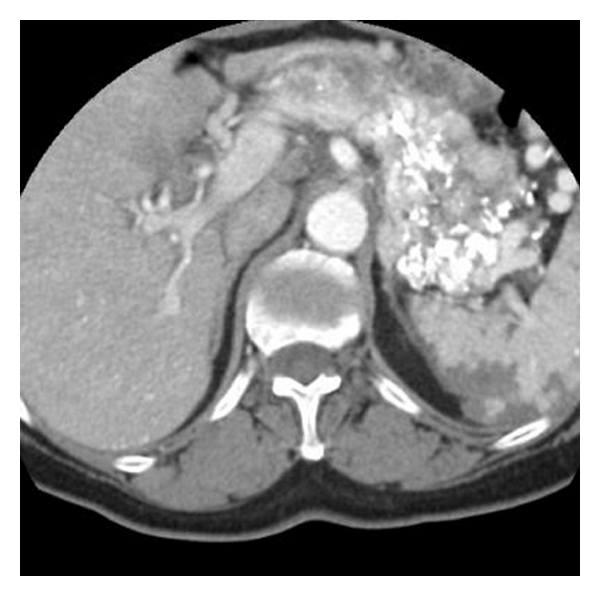
Contrast-enhanced axial CT scan through the pancreas after the embolization of the tumor shows multiple hyperdense embolic particles in the pancreatic tumor and also in the spleen and the right liver lobe.

A 64-year-old woman was referred to our Hospital for further evaluation of a symptomatic pancreatic mass, which was revealed on B-mode sonography. Two months before referral, she experienced persisting epigastric pain following a heavy meal. An upper gastrointestinal endoscopy revealed early gastritis and a positive CLO test for *Helicobacter pylori*. The patient received treatment for *H. pylori* eradication, improving her overall condition but failing to completely control the epigastric pain. Abdominal sonography was performed—for possible cholelithiasis—revealing a mass in the body-tail of the pancreas and thus the patient was referred to our institution for further investigation and management. She was diagnosed 6 years ago with multiple sclerosis complicated by cardiomyopathy for which she was under treatment. She had no history of trauma, pancreatitis, or other congenital abnormalities, and the only surgery she had undergone was right inguinal hernia repair. On physical examination, intense blepharoptosis was noted with generalized muscle weakness. Mild epigastric tenderness but with no definite mass was as well noted. Laboratory data were grossly normal apart from slightly elevated liver aminotransferases and *γ*-glutamyl transpeptidase, which were compatible with the sonographic finding of fatty liver infiltration. Serum amylase levels were within normal limits, as were serum Ca19-9 and CEA levels. The patient was tested negative for hepatitis B and C virus. She underwent abdominal CT and MRI scans, which in conjunction with the angiography that followed and revealed vascular distribution from the splenic artery, led to the diagnosis of pancreatic arteriovenous malformation (PAVM) (Figures 1, 2). On the CT images, the spleen was not oversized, while some varicose vessels with the more prominent one arising from the right gastroepiploic vein were noticed. At the same time, clinically there were no signs of portal hypertension. After explaining the severity and risks of a possible operative management, the patient refused any surgical intervention. Transcatheter arterial embolization was indicated and performed with acceptable results. Our objective for treatment, as for most of arteriovenous malformations, was to occlude as many of the shunts in the AVM as possible, so as to reduce the risk of portal hypertension, haemorrhage, and of course to alleviate pain, which was the initial clinical indication for intervention. A few hours after the intervention, the patient complained of a mild left upper quadrant pain which completely resolved within the next 24 hours. A slight increase in serum amylase (198 U/L) was noticed the next morning, which normalized in the next 2 days. There was no notable elevation of liver enzymes compared to the preinterventional levels, and the white cell count remained normal throughout the patient's admission. A day after the embolization, a repeat CT scan was performed. Embolic material was seen within the PAVM. A small number of embolic particles measuring only a few millimeters in size were seen scattered within the liver parenchyma and the spleen with concomitant small peripheral infarcts (Figure 3). The patient was closely observed and monitored after the embolization and only required mild pain relief. The patient after two years of followup remains asymptomatic, with only a few episodes of mild epigastric pain which may or may not be attributed to the PAVM. The small splenic infarcts have resolved without causing any complications such as persistent pain or abscess, which is seldom observed especially after small-sized infarcts as in our case. No signs of complications such as gastrointestinal bleeding or portal hypertension have been noticed.

## 3. Discussion

Pancreatic arteriovenous malformation is a very rare lesion representing about 5% of all arteriovenous malformations found in the gastrointestinal tract. PAVM was first described by Halpern in a patient with Rendu-Osler-Weber disease in 1968 [1]. Until today less than 80 cases of PAVM have been reported in the English-published literature including our patient. Pancreatic AVMs are classified into two categories according to their etiology: congenital which comprise 90% and acquired which comprise 10% [2]. Congenital AVMs arise from an anomalous differentiation in the rudimentary plexus of primordial blood vessels. Acquired AVMs are secondary to local inflammation, trauma, or tumors. The most frequently involved portion of the pancreas appears to be the head followed by the body and tail. Until today only about 6 patients have been presented with lesions occupying the entire organ. PAVM symptomatic patients usually present with abdominal pain or the possible life-threatening complication of gastrointestinal bleeding (in 50% of cases); the latter results from esophageal or gastric varices as a consequence of portal hypertension. In bleeding, blood may come to pass from the AVM into the pancreatic duct, from the AVM into the bile duct, from the intestinal mucosa when in direct contact to the AVM, and from a possible duodenal ulcer caused by the AVM [3]. Seldom, PAVM presents with portal hypertension or jaundice, while most cases are asymptomatic [4]. Portal hypertension develops when pancreatic AVMs grow progressively in size. This secondary portal hypertension due to hyperkinetic circulation can be resistant to treatment even after AVM resection, thus early surgical treatment of such lesions is suggested by some [2].

Pancreatic AVM has to be differentiated from other hypervascular neoplasms such as cystadenoma, cystadenocarcinoma, angiosarcoma, and islet cell tumor. Standard diagnostic approaches that have been used routinely in the last two decades include ultrasound and color Doppler sonography, computed tomography, magnetic resonance imaging, and angiography. New diagnostic tools are now utilized for the diagnosis of pancreatic arteriovenous malformations such as Multi-Detector Computed Tomography (MDCT) with specific image-processing techniques including Maximum Intensity Projection (MIP), Multi-Planar Reformation (MPR), and Volume Rendering (VR), which enable effective use of the large amount of the data obtained by MDCT [5]. MDCT which is widely used over the days can not only evaluate the local vascular system, but at the same time and in addition to that can give clear imaging of the surrounding organs [5].

Management of PAVM consists of surgical therapy and conservative therapy (arterial embolization, irradiation, portovenous shunt). Last but not least, a watch and wait policy with “no treatment at all” has been described in the literature. Nishiyama et al. suggested that complete cure is accomplished only by total extirpation of the affected organ or at least its involved part [2]. Koito et al. recommended that PAVM's should be treated with surgical resection whenever possible, taking under consideration the potential risk of portal hypertension [4]. In cases where surgery has a limited role which is mostly determined by size, location, and accessibility of the AVM, transcatheter arterial embolization may be the only alternative treatment [6]. It is therefore suggested that surgical resection or catheter embolization is indicated in some cases of PAVM, as long as they are accompanied by severe symptomatology [7]. However, as far as asymptomatic patients are concerned, there is no single established strategy leaving a watch and wait approach possible. In 2006, Kanno et al. presented 51 known cases of pancreatic AVM from world literature since 1968 in which 11 cases (22%) were asymptomatic out of which 7 received no treatment [8]. Appropriate therapeutic approach should follow a serious overall evaluation of the disease, taking under close consideration the size, location, and extend of the lesion as well as the possible risks of available management strategies.

##  Consent

Written consent of the patient was obtained before the information of her case was collected and she declared that it could be published or announced. All possible measures were taken that her identity is not revealed.
